# Interoceptive deficits in patients with obsessive-compulsive disorder in the time course of cognitive-behavioral therapy

**DOI:** 10.1371/journal.pone.0217237

**Published:** 2019-05-24

**Authors:** Dana Schultchen, Michael Zaudig, Till Krauseneck, Götz Berberich, Olga Pollatos

**Affiliations:** 1 Department of Clinical and Health Psychology, Institute of Psychology and Education, Ulm University, Ulm, Germany; 2 Klinikum Windach am Ammersee, Windach, Germany; 3 kbo-Isar-Amper-Klinikum, Munich-Haar, Germany; Institut d'Investigacio Biomedica de Bellvitge, SPAIN

## Abstract

Interoception is impaired in different psychiatric disorders and is also associated with emotions. Only one study could show a higher interoceptive accuracy (IAcc) in patients with obsessive-compulsive disorder (OCD). Based on the predictive coding system we assume contrary results, indicating a decreased IAcc in patients with OCD. So far, there is no study investigating the effect of cognitive-behavioral therapy on IAcc in patients with OCD. Therefore, we hypothesize that patients with OCD improve their IAcc during the time course of therapy compared to healthy controls. Twenty-six patients with OCD from the Psychosomatic Clinic in Windach were examined in the time course of cognitive-behavioral therapy. They were compared to 26 matched healthy controls. IAcc via the heartbeat perception task as well as questionnaire data (OCD-, depression- and anxiety symptoms) were assessed. Results showed that IAcc, OCD-, depression- and anxiety symptoms were attenuated in patients with OCD. Patients recovered in the time course of therapy regarding OCD-, depression- and anxiety symptoms. Interoceptive deficits did not change in the time course of cognitive-behavioral therapy. We demonstrated that IAcc is affected in patients with OCD and this deficit does not change during the time course of a standardized therapy. Future studies should investigate, whether an inaccuracy in perceiving one’s bodily signals constitutes a risk factor for relapse. Further, it could be examined if IAcc can be increased via self- and body focus interventions in patients with OCD.

## Introduction

Interoception is understood as the sensing and representation of signals concerning the internal state of the body [1,2]. There is a growing and ongoing interest in this construct because of the evidence that different mental disorders show impaired interoceptive processes and that mental processes are embodied (e.g., [3–8]). Consequently, an interplay between the mind and physiological states can be assumed.

Moreover, different research findings showed that an increased interoception is associated with an enhanced emotion perception, processing and regulation (e.g., [9–16]). These results are also supported by different emotional theories, indicating that the perception of internal bodily signals is essential for the development of emotions [17–19]. In concordance with these results and theories, neurological studies found similar patterns of brain activity during interoception and emotion elicitation, especially in the anterior insula [20–24]. To conclude, the perception of internal bodily signals, namely interoception, is central to the processing of emotions.

Based on the results of a disturbed interoception in mental disorders and the involvement of the insular cortex in the pathophysiology of obsessive-compulsive disorders (OCD) it is necessary to investigate the relationship between interoception and OCD. Its cardinal features are obsessions, i.e. unwanted and distressing thoughts or urges, and compulsions, i.e. repetitive behaviors that the person feels compelled to perform, typically with a desire to resist [25]. OCD symptoms can include excessive washing, constant checking (e.g., of door locks or electrical appliances), and recurrent, distressing thoughts, for example, of harming loved ones.

Song et al. [26] examined whether the volume of insular cortices of patients with OCD differed in regards to several symptom dimensions. They reported enlarged anterior and reduced posterior insular cortices in patients with OCD. The insular volumetric alterations were more significant in patients with OCD with predominant checking rather than cleaning symptoms when compared to healthy controls. Importantly, the authors suggested that insular morphometric alterations may influence the modulation of interoceptive processing in patients with OCD. So far, there is only one study investigating the relationship between interoception and OCD [27]. Authors reported that patients with OCD were better in interoceptive accuracy (IAcc) as measured by the tapping accuracy task, while less confident and aware of their interoceptive abilities. The authors interpreted their results as evidence that overactive monitoring in OCD extends to the sensing of internal bodily signals.

In contrast, we hypothesized that patients with OCD show a decreased IAcc level in comparison to matched healthy controls. This assumption is based on the theoretical ideas of predictive coding in the context of interoceptive processing (see, e.g. [28–30]). The idea is that people make individual perceptions based on their own working models, so-called priors. In this context, interoceptive signal processing provides an important source of information regarding the evaluation of such priors [28]. The logic of these models is that incoming sensory data are compared with internal models, i.e., the brain’s or “top-down” “predictions” about the body and the environmental causes that affect the organism, and thus can lead to “bottom-up” prediction errors (PE) that finally have to be minimized. Using a predictive coding formulation, the ability of interoceptive directed predictions to suppress otherwise inappropriate interoceptive PE allows the individual to infer the autonomic and emotional state of another. Having in mind typical OCD symptoms, patients with OCD regularly realize upcoming thoughts or urges to conduct a specific behavior (e.g., washing hands). When these thoughts are related to compulsive behavior they usually also experience a desire to resist such internal thoughts and urges, associated with anxiety or concern what could happen if they do not engage in compulsive behavior. Interoceptive signals are therefore a source of mistrust and anxiety, resulting in priors or beliefs that determine information from the body as not reliable and thus inadequate for reducing the PE. One possible solution to reduce bodily PE is to rely on other modalities like the visual channel as a compensatory strategy. This might contribute to a greater dependency on input from the external world to understand the causes of sensations evoked by emotionally salient stimuli which contribute to deviations in affective experience.

Former research provides some evidence for the assumption above. For example, empirical data report that patients with OCD show tendencies to doubt their feelings and internal states (e.g., [31,32]). More specific, Shapiro [32] described that patients lost the ability to access internal states and instead use external indicators (e.g., biofeedback) to make conclusions about these states. For example, when patients with OCD had to answer the question whether they had fallen in love with another person, they did not trust their feelings. Instead, they checked external indicators (such as general rules or norms), which represent the feeling of falling in love [32]. Consequently, Shapiro [32] would argue, that patients with OCD fall in love when the partner shows all the right attributes but not because of a feeling.

That these external cues or interpretation are important for patients with OCD is supported by the research of Lazarov and colleagues [33,34]. Based on the theory of Liberman and Dar [35], they assume that patients with OCD display a decreased sense of subjective confidence and this leads to a higher focus on external “proxies” (i.e., opportunities to prove internal states) to compensate their deficits regarding inner subjective experience [34]. Results indicate that patients with OCD perform poorly on tasks with a focus on inner subjective feelings or states, in comparison to healthy controls. However, if they have the opportunity to use external proxies (e.g., biofeedback), they improve their task performance compared to healthy controls. Lazarov and co-workers [33] observed similar results with a non-clinical sample differentiating between low and high OCD tendencies. Furthermore, research showed that participants with high OCD tendencies relied on false feedback from biofeedback instead of their own internal state of relaxation [33]. This tendency could also be confirmed in the clinical sample [34]. We therefore follow that patients with OCD exhibit disturbances in the complex integration of information from interoceptive and exteroceptive channels resulting in severe deviations of affective experience of oneself and others. The perception of interoceptive signals is assumed to be reduced. One of the reason could be that typical OCD symptoms such as the occurrence of intrusive thoughts as well as the distrust of patient’s attention, perception and senses might lead to a distraction from heart rate counting or other mindfulness processes with a focus on internal bodily signals [36–40].

To sum up, interoceptive alternations might affect priors that determine information from the body as not reliable and thus are deemed inadequate for updating the prior and reducing the predictive error. As suggested by Lazarov and colleagues [33,34], one possible solution to reduce bodily predictive error from the view of a patient with OCD is to rely on other external modalities such as biofeedback as a compensatory strategy. Based on these findings, we will first investigate our assumption that IAcc, which is defined as the objective performance marker for interoception, is diminished in OCD. It should be noted that this hypothesis is not in line with the data of Yoris et al. [27]. Second and not investigated until now, we will examine the effect of cognitive-behavioral therapy for OCD on IAcc. Based on the assumed positive effects of cognitive-behavioral therapy, we hypothesized that patients with OCD would improve their IAcc during the time course of therapy compared to healthy controls. We also assessed OCD-, depression- and anxiety symptoms to identify possible working mechanism of the therapy, as well as potential factors influencing IAcc throughout the course of therapy.

## Materials and methods

### Participants

Twenty-six patients with OCD (14 males; all Caucasian ethnicity) were recruited from the Psychosomatic Clinic Windach am Ammersee after admission as well as after agreement of therapist and patient. Their diagnoses were determined based on the International Classification of Diseases by using semi-structured clinical interviews, which were conducted by senior staff members. Mean age in the OCD group was 28.6 years (*SD* = 7.2). Exclusion criteria were comorbidity with other psychotic disorders and intake of beta-blockers. Seventeen patients received antidepressant medication (SSRIs, tricyclic antidepressants), one took neuroleptic medication and one benzodiazepine; nine patients did not receive psychotropic medication. Additionally, other drugs were taken regularly, including four patients receiving thyroid hormones, one contraceptive and two asthma medication (partially on demand). Five patients were adjusted during the therapy. In another two patients, the depressed medication was started. Comorbidities included mostly depression (*n* = 13), followed by anorexia nervosa (*n* = 2), panic disorder (*n* = 1), social phobia (*n* = 1) and somatic disorder (*n* = 1).

All patients took part in the cognitive-behavioral inpatient therapy program of the clinic with a particular focus on emotion-, mindfulness- and body-related therapy. The program consists of an intensive group- and individual therapy elements. The central part includes a 2-week period of exposure therapy (flooding) towards the trigger of obsessive or compulsive symptoms and systemic interventions. Additionally, the therapy pays attention to maladaptive emotional processes, the systemic context, mindfulness meditation (i.e., breathing meditation and body scan), schema therapy as well as body psychotherapy. If needed, patients will also receive medication, which mostly includes antidepressants (such as SSRI). The whole therapy lasts between 8 and 10 weeks.

Twenty-six healthy controls were recruited by advertisement in local newspapers and from staff or students at the Ulm University. They were matched according to age, sex, and educational background. Controls had a mean age of 26.5 (*SD* = 5.6). None of them took medication (except contraceptives), had past or current psychiatric disorders or severe somatic illness as assessed by an anamnestic questionnaire. Both groups did not differ significantly in age (*t*(50) = 1.16, *p* = .25). Patients and controls received a compensation of € 20 for their participation and were only included in the data analyses if they participated in all three measurements and patients with OCD successful finished their therapy (inclusive of flooding).

### Design

Experiments were conducted by the Declaration of Helsinki with the approval of the Institutional Review Board of Ulm University. All participants were informed verbally and in writing about the study by staff and received written information about the experiment. Next, participants had to sign the informed consent form to take part in the study. They were tested individually in a separate, quiet room of the clinic. Controls were examined at the laboratories of the Clinical and Health Psychology Department at Ulm University. To capture changes in different variables throughout the time course of therapy, patients were investigated three times based on the therapy-process, i.e., at the beginning (T1), after 4–6 weeks (T2) and at the end after flooding/exposure therapy (after 8–10 weeks, T3). Patients and controls started to fill out the questionnaires prior to each testing session. In the following, we will refer to a subset of data including OCD-, depression- and anxiety symptoms. Current OCD severity was assessed using the Yale-Brown Obsessive Compulsive Scale (Y-BOCS; [41,42]). Results were only reported for patients with OCD because it is not appropriate to use the scale in a healthy sample. Other assessments were the Beck Depression Inventory (BDI; [43]) to assess depression symptoms and the trait version of the Spielberger State-Trait Anxiety Inventory (STAI; [44]) to quantify the anxiety level.

It should be noted that the questionnaires were integrated to identify the possible confounding character. After completing the questionnaires, the physiological measurement of IAcc by the heartbeat perception task by Schandry [45] were administered. Other paradigms were used that will be not reported here. Each session lasted about 45 minutes. The same experimental procedure was applied for all three testing points. Controls were also tested three times using a comparable timetable.

### Instruments

#### Yale-brown obsessive-compulsive scale

The Y-BOCS [41,42] assesses the severity and symptoms in patients with OCD with ten items. For this study, the self-report version of the Y-BOCS was used to measure obsessive thoughts and obsessive actions on a five-point Likert scale, ranging from 0 (= *none*) to 4 (= *extreme*). Mean scores vary between 0 and 40. Studies investigating the psychometric criteria of this self-report tool indicate excellent reliability and validity [46].

#### Beck depression inventory

The BDI [43] is a widely used self-report measure of depressive symptoms regarding the last week, including 21 items. These items are evaluated on a four-point rating scale, ranging from 0 (= *not at all*) to 3 (= *always*). The BDI provides scores between 0 and 63. The internal consistency differs between 0.84 ≤α ≤ 0.94 for clinical and non-clinical samples (e.g., [47–52]).

#### State-trait anxiety inventory

The self-reported and well-validated STAI [44] quantifies the anxiety level of individuals. For this study, we only used the 20-item trait version to measure the dispositional anxiety level. Participants have to rate their dispositional feeling on a four-point Likert-type scale (1 = *almost never* to 4 = *almost always*).

#### Heartbeat perception task

The heartbeat perception task by Schandry [45] was used to assess IAcc. Therefore, cardiac activity was recorded using the mobile heart frequency monitor RS800CX (Polar Electro Oy, Kempele, Finland) with electrodes placed in a belt and attached at the chest. The RS800CX is easy to use. Moreover, it is a non-invasive and -reactive recording of inter-beat-intervals whose validity and reliability compared to alternative ECG measurement devices is established. It was used similarly in previous studies [53,54]. The heartbeat perception task consisted of a three-interval block, including 25, 35 and 45 seconds as proposed by Schandry [45], presented in a random order. To get familiar with the task, a training interval of 10 seconds was included. The beginning and the end of each trial were indicated by the experimenter. During heartbeat counting, participants were instructed not to take their own pulse or attempt to use other forms of manipulation for counting their heartbeats. They did not receive any information about the duration of the counting phases or the quality of their performance. Directly after the end of the interval participants had to report their counted heartbeats verbally. IAcc was calculated as the mean heartbeat perception score according to the following transformation of the three intervals:

$$\frac{1}{3}\sum (1-\frac{\left(|recorded\;heartbeats-counted\;heartbeats|\right)}{recorded\;heartbeats}$$

IAcc scores range from 0 to 1. Higher scores indicate smaller differences between the counted and recorded heartbeats and consequently a better IAcc.

### Data analyses

Data analyses were performed using the program SPSS (version 24). Referring to BDI, STAI, and IAcc, repeated measures ANOVAs were calculated with the factors Time (T1, T2, T3) and Group (patients with OCD/healthy controls). For the Y-BOCS only time effects will be presented focusing on the patients with OCD. Statistical significance levels reported corresponding p-values less than .05, respectively. In the results section, uncorrected F-values are reported together with the Greenhouse-Geiser epsilon values and corrected degrees of freedom. Effect sizes (η^2^, ε) were also reported. Additional analyses were performed to investigate the influencing effect of medication and comorbidities on OCD-, depression- and anxiety symptoms as well as IAcc. Further, we also integrated depression and anxiety symptoms as co-variates in the repeated measure ANOVA for the IAcc analyses. Lastly, one-sided Pearson correlation analyses were conducted for IAcc and clinical measures (OCD, depression-, and anxiety symptoms).

## Results

All descriptive data of IAcc and OCD-, depression- and anxiety symptoms separated by patients and healthy controls are represented in Table 1. As shown in this table, OCD-, depression- and anxiety symptoms decrease during the time course of cognitive-behavioral therapy in the OCD sample. Furthermore, there is a descriptive increase in IAcc over time for patients with OCD. Moreover, patients have a decreased IAcc and more depressive- and anxiety symptoms in comparison to the healthy controls. These differences are significant for most group comparison at the different measurement points. Inferential statistic results will be described in more detail in the following sections.

**Table 1:** Descriptive data for OCD-, depression- and anxiety symptoms and interoceptive accuracy contrasting patients with OCD and healthy controls.

	*OCD*		*Controls*	
	Mean (SD)	Range	Mean (SD)	Range
*OCD symptoms*				
T1	21.27 (8.13)	9–38		
T2	18.85 (7.34)	9–40		
T3	13.08 (6.94)	2–36		
*Depression*				
T1	19.31 (8.77)	5–34	3.42 (2.16)	0–7
T2	18.65 (7.84)	4–36	2.58 (1.84)	0–6
T3	15.54 (8.56)	4–36	3.08 (2.13)	0–7
*Trait anxiety*				
T1	49.46 (5.67)	38–61	45.23 (4.05)	37–52
T2	48.46 (4.65)	41–58	45.12 (4.60)	37–54
T3	46.85 (5.40)	38–58	44.04 (4.75)	36–55
*Interoceptive accuracy*				
T1	.60 (.17)	.35 - .92	.75 (.17)	.39 - .97
T2	.62 (.15)	.27 - .91	.75 (.19)	.30 - .97
T3	.66 (.17)	.26 - .92	.76 (.16)	.39 - .97

Note: OCD = obsessive-compulsive disorder; SD = standard deviation. T1 = baseline, T2 = 4–6 weeks after therapy onset, T3 = 8–10 weeks after therapy onset.

### Obsessive-compulsive symptoms (Y-BOCS)

For the Y-BOCS only results of the OCD group in the time course were reported. Descriptive data showed a decrease regarding the OCD symptoms from T1 to T3 (see Table 1). The repeated ANOVA revealed significant effects of Time *F*(1.50, 37.42) = 20.65; *p* < .001; *η*^*2*^ = .82; *ε* = .75), indicating that severity of compulsive thoughts and actions significantly decreased over time in patients with OCD. Additional analyses to test the impact of medication or comorbidities showed that the significant time effect is not influenced by medication (*F*(1, 23) = .23; *p* = .639) or comorbidities (*F*(1, 23) = 1.07; *p* = .312).

### Depression (BDI)

The repeated measures ANOVA revealed significant main effects of Group (*F*(1, 50) = 97.65; *p* < .001; η^2^ = .66; *ε* = 1.00), Time *F*(1.65, 82.60) = 4.60; *p* = .018; *η*^*2*^ = .09; *ε* = .83), and, importantly, an interaction Time x Group (*F*(1.65, 82.60) = 4.40; *p* = .021; *η*^*2*^ = .08; *ε* = .70). Consequently, the BDI also significantly decreased over time for patients with OCD in comparison to healthy controls (see Table 1). Additional analyses showed no influence of medication (*F*(1, 23) = .01; *p* = .944) or comorbidities (*F*(1, 23) = 2.81; *p* = .108) in the OCD sample.

### Trait anxiety (STAI)

Regarding trait anxiety we observed similar effects for Group (*F*(1, 50) = 8.19; *p* = .006; *η*^*2*^ = .15; *ε* = .87) and Time *F*(2, 100) = 6.88; *p* = .002; *η*^*2*^ = .13; *ε* = .67). However, these effects couldn’t confirm for Time x Group (*F*(2, 100) = .93; *p* = .399). Results reflect a decrease in trait anxiety over time for patients with OCD only (see Table 1). Moreover, no impact of medication (*F*(1, 23) = 1.18; *p* = .289) nor comorbidities (*F*(1, 23) = .39; *p* = .540) could be found for the OCD group.

### Interoceptive accuracy (heartbeat perception task)

**Fig 1:**
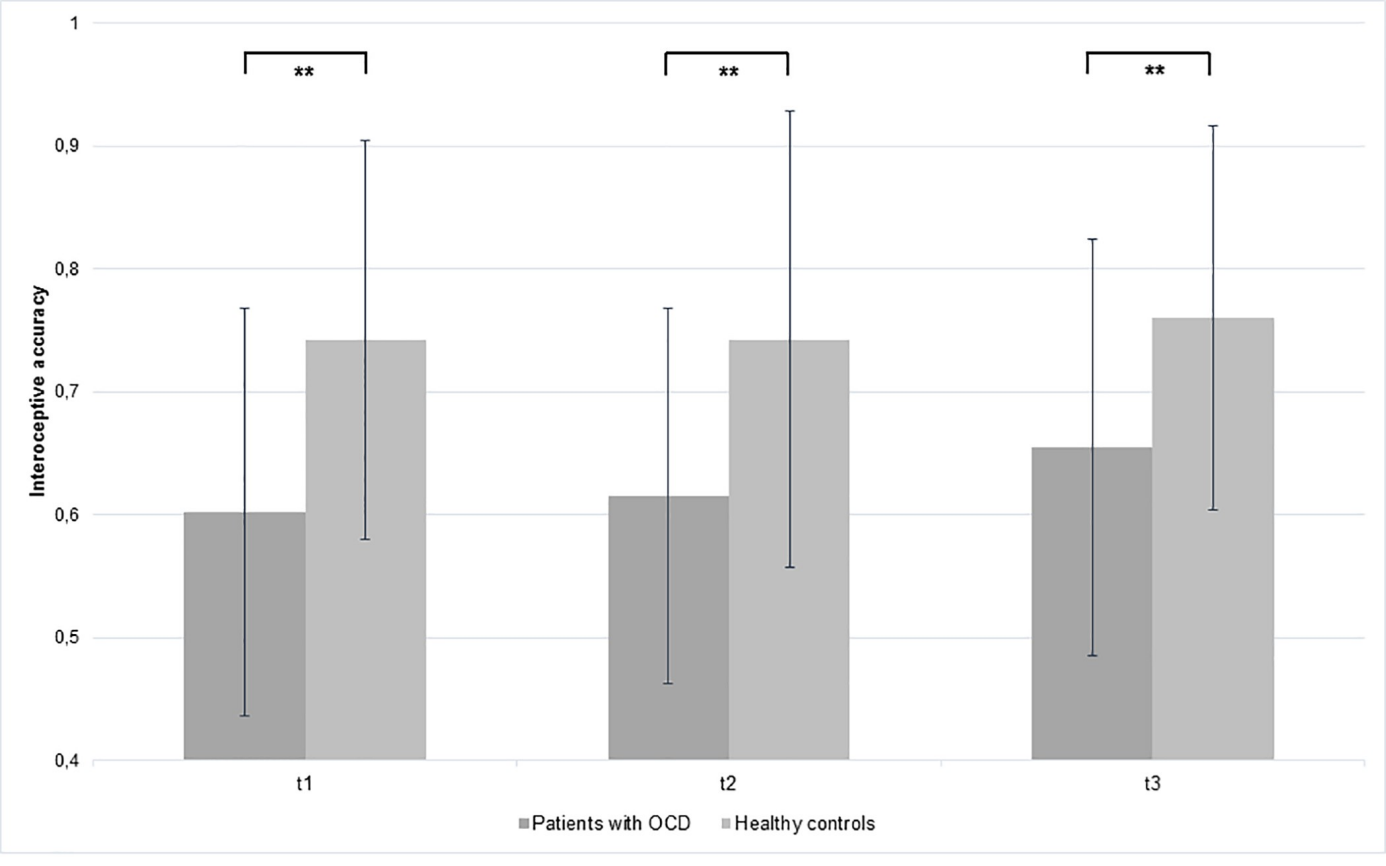
Mean and standard deviation of interoceptive accuracy in patients with OCD compared to healthy controls in the time course of therapy (p < .01 = **).

The mean and standard deviation obtained heartbeat perception scores for both groups are summarized in Fig 1 and Table 1 depicting T1, T2 and T3. We observed a significant main effect of Group (*F*(1, 50) = 10.94; *p* = .002; *η*^*2*^ = .17; *ε* = .87), indicating that patients with OCD had a lower IAcc (average score for all three measurements 0.63) at all three measurement points as compared to healthy controls (average score for all three measurements 0.74). No other main or interaction effect occurred (Time: *F*(2,100) = 1.64; *p* = .200; Time x Group: *F*(2,100) = 0.41; *p* = .668). It should be noted, that there was a descriptive higher improvement of IAcc for the OCD group (mean T1 = .60 (*SD* = .17), mean T2 = .62 (*SD* = .15), mean T3 = .66 (*SD* = .17)) compared to the healthy controls (mean T1 = .74 (*SD* = .16), mean T2 = .74 (*SD* = .19), mean T3 = .76 (*SD* = .16)). Similar to the results before, we did not find any influence of medication (*F*(1, 23) = .35; *p* = .560) or comorbidities (*F*(1, 23) = .07; *p* = .796) for the OCD group. Moreover, we integrated depression and anxiety symptoms as co-variates, which did not change the main effect of Time *F*(2,96) = .13; *p* = .882. Further, it could be shown that there is no influence of depression (*F*(1, 48) = 1.26; *p* = .268) nor anxiety (*F*(1, 48) = .51; *p* = .480) regarding the results in both groups.

### Correlation analyses between interoceptive accuracy and clinical measures

Additional correlation analyses were conducted with both OCD patients and healthy controls at T1 focused on the relationship between IAcc and clinical measures, including OCD-, depression- and anxiety symptoms. It was shown that a reduced IAcc was related to more OCD symptoms (*r* = -.451; *p* < .001). Regarding the depression and anxiety symptoms the correlation was marginal significant (*r*_Depr_ = -.213; *p* = .06; *r*_Anx_ = -.211; *p* = .06), indicating a similar direction. Performed separate analyses for patients and, in the next steps, with healthy controls revealed no significant relationships between IAcc and clinical measures (*p* > .08).

## Discussion

To the best of our knowledge, these are the first empirical data demonstrating that IAcc is attenuated in OCD and that these interoceptive deficits do not change in the time course of cognitive-behavioral therapy. Nonetheless, there were definite improvements in OCD-, depression- and anxiety symptoms for patients with OCD. Additionally, we also showed negative associations between IAcc and OCD-, depression- and anxiety symptoms.

The idea that interoceptive processes might be affected in OCD was proposed mainly based on research concerning brain activities in the insula as well as the dysbalance of internal and external channels in patients with OCD. Song and co-workers [26] demonstrated altered insula activation patterns in OCD. Supporting the authors’ hypotheses that insular morphometric alterations may influence the modulation of interoceptive processing in patients with OCD, we could demonstrate that IAcc is reduced in patients with OCD. Furthermore, prior studies observed that patients with OCD have tendencies to not trusting in their internal states and feelings (e.g., [32,31]). Instead, patients seem to compare their assumptions of internal states with external indicators like general rules or norms. This postulation could be supported by a study of Lazarov and colleagues [33,34], showing that patients with OCD improved their performance when they had the opportunity to use biofeedback to prove their internal states. Conversely, Yoris and co-workers [27] demonstrated that patients with OCD show an increased IAcc in comparison healthy controls and patients with panic disorders. One reason for different results could be that they used the tapping accuracy task of Canales-Johnson et al. [55] instead of the heartbeat perception task [45]. For the tapping task, participants were instructed to firstly tap with their dominant-hand in synchrony with an audio-recorded heartbeat and secondly to their own heartbeat. For the IAcc score, time differences between the R-Peaks and the motor-taps incorrect answers were calculated. A smaller difference between both indicates a better IAcc. In their discussion, Yoris et al. [27] suggest replicating their results with non-motor interoceptive tasks such as the heartbeat perception task. We used the heartbeat perception task in our study. In comparison to tapping with the hand to different heartbeats in the tapping accuracy task, participants were instructed to focus on their heartbeat and to silently count it over three different time intervals. A smaller difference between recorded heartbeats via ECG and counted heartbeats indicate a better IAcc. It should be noted that there are different focuses in the tapping and heartbeat perception task. Whereas the tapping accuracy task includes the focus on interoceptive and exteroceptive signals (heartbeat and tapping), the heartbeat perception task only concentrates on interoceptive signals. To summarize, we could not show similar results in the heartbeat perception task, but in our opinion, these are two different measurements of IAcc, which make the comparison of the findings of the two studies difficult.

The observed interoceptive alternations did not change in the time course of cognitive-behavioral therapy in patients with OCD. So far, there are no other studies that also examine changes in IAcc during the time course of therapy. The present findings are of high importance for the pathophysiology of OCD. A reduced IAcc might constitute a risk factor for a more stressful everyday life as abilities to regulate one’s emotions are needed and are associated with more positive well-being [56]. Another point is that this form of cognitive-behavioral therapy does not focus on the perception of internal states. In a study of patients with anorexia nervosa, also only a trend regarding the improvement in IAcc was observed in the same clinic with the same therapy [57]. Consequently, it could be useful to develop an intervention focusing only on internal bodily signals and the self (e.g., looking in the mirror). There is evidence for positive effects on interoceptive ability when applying this kind of methods (e.g., [58,59]). Based on the findings of Lazarov and colleagues [33,34], another interesting point for future studies would be the inclusion of a biofeedback intervention with a reduction of the biofeedback over time to reduce the reliance on external stimuli’s and to increase the focus on internal signals step by step.

Different strengths and limitations of this study should be noted. An advantage of the study is that participants were matched according to age, sex, and educational background. Additionally, this is the first study investigating IAcc during the time course of cognitive-behavioral therapy. Potential limitations of this study refer to the fact that we used an OCD sample that was heterogeneous in terms of symptoms and comorbidities, as well as medication use. For example, comorbidities like depression, which highly occurs in the present sample as comorbidity, could be an influencing factor on observed results. This assumption is based on different studies, indicating an impaired IAcc in depressive participants (e.g., [3,60]). Though we addressed this issue and found no influence, the effects of medication on autonomic measures cannot be ruled out when using only small numbers of patients in each subgroup. Additional analyses with the inclusion of medication and comorbidities as co-variates showed no influencing effect regarding the reported results with OCD-, anxiety- or depressive symptoms nor IAcc in the OCD sample. Nevertheless, these results should be interpreted with caution due to the small sample size of OCD patients. Furthermore, Fischer et al. [58] found that a body scan training improved IAcc. Consequently, mindfulness should also be included in future studies as another confounding variable.

## Conclusions

To conclude, our study highlighted a decreased IAcc in patients with OCD and the observation that these deficits do not seem to improve during the time course of cognitive-behavioral therapy. Future studies should use longer follow-ups and additionally examine more levels of interoceptive signal processing such as metacognitive confidence (i.e., interoceptive awareness) or subjective awareness (i.e., interoceptive sensibility). Besides, it could be interesting to differentiate between obsessive-compulsive thoughts and behaviors in future studies. It could also be useful to integrate another psychiatric control group (e.g., panic disorder) as in the setting of Yoris et al. [27]. Lastly, using methods in therapy that can improve IAcc (e.g., body scan; [58]) should be considered to be of high relevance.
